# Use of Low-Cost Acquisition Systems with an Embedded Linux Device for Volcanic Monitoring

**DOI:** 10.3390/s150820436

**Published:** 2015-08-19

**Authors:** David Moure, Pedro Torres, Benito Casas, Daniel Toma, María José Blanco, Joaquín Del Río, Antoni Manuel

**Affiliations:** 1 Centro Geofísico de Canarias, Instituto Geográfico Nacional, C/La Marina 20, 38001 S/C Tenerife, Spain; E-Mails: patorres@fomento.es (P.T.); bcasas@fomento.es (B.C.); mjblanco@fomento.es (M.J.B.); 2 SARTI Research Group. Electronics Department, Universitat Politècnica de Catalunya (PUC), Rambla Exposició 24, Vilanova I la Geltrú, 08800 Barcelona, Spain; E-Mails: daniel.mihai.toma@upc.edu (D.T.); joaquin.del.rio@upc.edu (J.D.R.); antoni.manuel@upc.edu (A.M.)

**Keywords:** multiparametric system, volcano monitoring, ARM, Linux system, raspberry PI

## Abstract

This paper describes the development of a low-cost multiparameter acquisition system for volcanic monitoring that is applicable to gravimetry and geodesy, as well as to the visual monitoring of volcanic activity. The acquisition system was developed using a System on a Chip (SoC) Broadcom BCM2835 Linux operating system (based on DebianTM) that allows for the construction of a complete monitoring system offering multiple possibilities for storage, data-processing, configuration, and the real-time monitoring of volcanic activity. This multiparametric acquisition system was developed with a software environment, as well as with different hardware modules designed for each parameter to be monitored. The device presented here has been used and validated under different scenarios for monitoring ocean tides, ground deformation, and gravity, as well as for monitoring with images the island of Tenerife and ground deformation on the island of El Hierro.

## 1. Introduction

Volcanic eruptions are phenomena that can significantly affect populations in the immediate surroundings of the point of eruption and those located at greater distances (e.g., flight disruption caused by ash clouds). These natural phenomena are usually preceded by a reactivation phase in which measureable variations in parameters occur [1]. Volcanic monitoring provides the necessary data and, together with knowledge of past activity, can be used to predict future behaviour (albeit with implicit uncertainties) [2].

These factors justify the importance of using appropriate methods in volcano surveillance to better anticipate eruptions, thereby minimizing the risk for population and possible economic disruption. Volcanic areas are usually remote and inaccessible and only 30% of active volcanoes have instrumentation to monitor their activity [3], because of that, it is advisable to use low cost and low power consumption instrumentation for volcano monitoring.

Until recently, instrumentation dedicated to study the volcanic activity was specially designed for each monitoring technique: seismology, geodesy and geochemistry, with several problems to keep it continuously running, for example: high power consumption, data incompatibility and different communications systems [4,5]. As a result of these problems, data series are incomplete, and intercomparison, which is the basis of a multidisciplinary science like volcanology, is also difficult [6,7,8].

That situation has changed in the last few years with the development of new instruments using chip technologies allowing for real-time monitoring [9]. These devices have improved their power consumption [10], taking advantage of renewable energies and wide variety of ways of systems available for transferring data [11,12,13,14], but also using standard protocols [15,16,17].

Proper monitoring of volcanic activity is chiefly carried out using a combination of geophysics, geodesy, and geochemistry. Nowadays, new techniques such as gravimetry [18], geoelectricity [19,20] and geomagnetism [21] complement the by-now classic surveillance techniques and ensure a better understanding of volcanic activity in particular areas. Despite being general-purpose in type, the first applications of the system described in this paper employed geodetic and gravimetric techniques.

Microgravimetry is used to quantify geophysical density changes below the Earth’s surface and studies that employ this technique are usually conducted over a period of days or weeks (continuous recording is possible). They are repeated from periodically in different parts of the study area and yield variations in gravity measurements that are then used to characterize the state of the system [22]. With the development of continuous real-time measurement systems, temporal resolution of gravity measurements has been improved [23]. Furthermore, since gravimeters are highly sensitive instruments capable of measuring variations in gravity of just a few µGal (as shown in Table 1), they are very useful for monitoring volcanic activity. However, this sensitivity makes it essential that meteorological parameters are recorded simultaneously in order to ensure correct gravity measurements [24].

**Table 1:** Gravimeters used in volcanic monitoring.

Manufacturer	Model	Range	Resolution
LaCoste Romberg	G	7000 mGal	5 uGal
Micro-g LaCoste	gPhoneX	7000 mGal	0.1 uGal
ZLC Corporation	Burris	7000 mGal	1 uGal
Scintrex	CG5	8000 mGal	1uGal

Changes in gravity as a precursor of volcanic activity have been detected in numerous volcanic areas [25,26] and the way in which vibrations of paroxyseismic explosions affect gravimeters has been studied in many locations [27].

The most commonly used devices in continuous monitoring of ground deformation are GPS receivers and tiltmeters. Different designs of tiltmeters exist, each with their respective advantages and disadvantages in terms of cost, complexity, sensitivity, and ease-of-use (Table 2). They can measure Earth tides from 0.1 µrad to 10^−3^ µrad. The great advantage of this type of sensor is that its output is directly proportional to the slope of the land produced by volcanic activity and, unlike GPS receivers that require complex post-processing [28], the data acquired remotely from tiltmeters can be analyzed in real time.

**Table 2:** High-precision tiltmeters.

Manufacturer	Model	Type	Resolution
Jewell Instruments	LILY	Borehole	<0.01 µrad
Sherborne Sensors	T235	Platform	<0.1 µrad
Singer Instruments	TS series	Platform	<0.1 µrad
Altheris Sensors & Controls	AILSO series	Borehole	<0.1 µrad

The information provided by tiltmeters is very useful—above all when combined with other techniques—for characterizing phenomena such as intrusive dikes [4] and fault motions [29]. It is very important to have long series (years) of continuous data for these phenomena to be able to establish a reliable baseline level [30]. Based on large data series, it is sometimes possible to establish a relationship between the measured signals and the types of eruption [31].

Tide gauges are devices that measure the distance between sea level and a point with known geodetic coordinates; nevertheless, due to their precision, they can also be used to measure vertical displacements (Table 3). In the case of volcanoes located near the sea, a set composed by a tide gauge and a GPS is a useful tool for measuring vertical ground motion due to volcanic activity [32,33]. As well, a volume of water can be used as a natural tiltmeter [34,35]. In order to detect centimetric vertical variations, the influence of the meteorological variables should be removed from the tide-gauge registers. In addition, reference stations should be deployed outside the zone of deformation [36].

**Table 3:** Sensors used for tide measurements.

Manufacturer	Model	Type	Precision
Vega	VegaPuls 62	Radar	±2 mm
Seba Hydrometrie	SebaPuls 30	Radar	±3 mm
OTT Hydromet	OTT Thalimedes	Float	±2 mm

Finally, although essential at all times, the visual monitoring of a volcano is especially vital when it is evidently active and provides additional information for assessing its status. Examples of the importance of monitoring volcanic activity using images can be found in works on the characterization of highly explosive eruptions [37] and in studies of the relationship between the data recorded by other techniques and their effect on the Earth’s surface [38].

## 2. Low-Cost Acquisition System with Embedded Linux

In the last few years different systems with embedded Linux devices have been used for volcano monitoring [39,40,41]. Most of them are based on minority platforms with a limited developer’s community, which means less documentation and lack of examples for using its hardware and peripherals. Nowadays, there is a boom on the development of systems with an embedded Linux unit, originally intended for educational purposes, being the most important: Arduino Yun [42], Banana Pi [43], BeagleBone Black [44], Intel Galileo [45] and Raspberry Pi [46]. These systems have allowed the growth of different development communities, where is especially emphasized the case of the Raspberry Pi, whose community is one of the most important and numerous, allowing easier and faster learning than possible with other platforms. This implies, on the one hand, that a lot of documentation about the hardware and software is provided, with a lot of code examples and libraries. On the other hand, it allows regular software updates, bug fixes and security patches of the Linux system.

Table 4 shows a comparison between five of the main platforms with embedded Linux, all of them suitable for the development of data acquisition systems. In our case, we choose the Raspberry Pi platform because of three fundamentals factors: the first one is the largest community behind this platform, the number of developers is so great that allows fast development of applications because of the great amount of code examples and libraries; the second one is the employment of a platform that allow us to use standard communication protocols and standard data format, that permits an easy and fast intercomparison between data from different techniques in the volcanic monitoring network; the last one is the accessibility and price of the hardware, it can be bought on international electronic distributors, local electronic stores and shopping malls.

Finally, much of the equipment used in volcano monitoring is installed in remote areas using batteries and solar panels as power sources. It is therefore desirable that the power consumption of the equipment be the lowest possible, but due to the fact the consumption of the systems shown are similar, this isn’t a feature that determine their selection.

The low-cost acquisition system described in this study is based on a small (credit-card size) computer that offers multiple communication protocols such as Inter Integrated Circuit (I2C), Serial Peripheral Interface (SPI), Universal Asynchronous Receiver-Transmitter (UART), Universal Serial Bus (USB), and Ethernet. We built a data-acquisition system with circuitry designed specifically to acquire signals at 16-bit resolution with low noise [47]. The acquisition system has a real-time clock for situations in which there is no GPS or where the Network Time Protocol (NTP) cannot be used due to a lack of connectivity. We also developed all the different software applications for control, visualization, storage, and data display. Based on a Linux embedded system, the applications running in this acquisition system allow users to configure and display the data being recorded in real time via a web page or instant messaging software and thus to track activity instantly from almost anywhere.

**Table 4:** Comparison between some Linux platforms. Prices were obtained from an international electronic distributor while the power consumptions of each device are obtained from the respective manufacturers’ web pages.

	Arduino Yun	BeagleBone Black	Banana Pi	Intel Galileo	Raspberry Pi B
**SoC**	Atheros AR9331	TI AM3358	Allwinner A20	Intel Quark X1000	Broadcom BCM2835
**CPU**	MIPS32 24K and ATmega32U4	ARM Cortex-A8	ARM Cortex-A7	Intel X1000	ARM1176
**RAM**	64 MB	512 MB	1 GB	256 MB	512 MB
**Interfaces**	WiFi, USB, Ethernet, UART, SPI, I2C	USB, Ethernet, UART, SPI, I2C	USB, Ethernet, UART, SPI, I2C	USB, Ethernet, UART, SPI, I2C	USB, Ethernet, UART, SPI, I2C
**Camera Interface**	No	Yes	Yes	No	Yes
**Video output**	No	HDMI	HDMI/Comp	No	HDMI/Comp
**Power supply**	5 V	5 V	5 V	5 V	5 V
**Power consumption**	1.35 W	1.6 W	1.15 W	2.5 W	1.8 W
**Price**	67.36 €	51.99 €	43.23 €	45.51 €	20.67 €

### 2.1. Hardware

The basic function of the system is: (i) the digital conversion of the acquired analog signal using 16-bit resolution; (ii) the on-board storage of this data and (iii) its transmission and display. The sampling rate, the number of channels to be acquired, and other information regarding the parameters being measured are configured mainly from a purpose-built website.

For the development of the data-acquisition system, a Raspberry Pi^TM^ microcomputer (Table 5) on an ARM^TM^-embedded processor with the Raspbian operating system (based on Debian Linux) was chosen for its low cost and consumption. Given that it supports different GNU/Linux operating systems, it is ideal for developing a real-time measurement system and facilitates the development of applications across multiple communication protocols such as User Datagram Protocol (UDP), Transmission Control Protocol (TCP), File Transfer Protocol (FTP), and Secure Shell (SSH) and Secured File Transfer Protocols (SFTP). Moreover, since the programming uses high-level languages it is also very easy to develop applications for data manipulation.

This acquisition system consists mainly of a low-noise 16-bit A/D converter from Analog Devices (Norwood, MA, USA) and a real-time clock (RTC) from NXP Semiconductors (Eindhoven, Netherlands), which is necessary if the system loses its Internet connection and cannot be synchronized via the NTP protocol. Currently, we are testing a new version based on a 5-channel A/D converter with 24-bit resolution.

**Figure 1:**
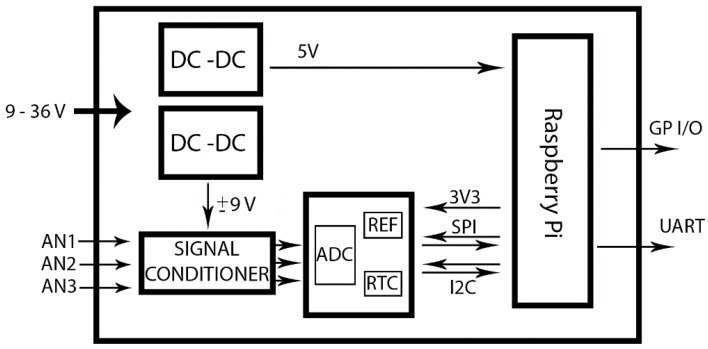
General schema.

Using different sensors, an electronic signal conditioning was developed that can adapt the acquisition circuit by adjusting the measurement signal to the input range of the A/D converter, and can filter the acquired signal (Figure 1). The applications developed based on this acquisition system are described below.

**Table 5:** Main Features of Raspberry Pi.

Raspberry Pi Model B	
SoC	Broadcom BCM2835 (CPU,GPU,DSP,SDRAM,USB)
CPU	ARM 1176JZF-S 700 MHz (ARM11)
Instructions	RISC 32 bits
SDRAM	512 MiB
USB 2.0	2
Video output	HDMI,RCA,DSI
Storage	SD/MMC
Network connectivity	10/100 Ethernet
Peripherals	GPIO,SPI,I2C,UART
Power consumption	200 mA @ 12V

#### 2.1.1. Gravity Measurement System

The sensor used for the gravimetric system was a LaCoste & Romberg (LCR, Lafayette, CO, USA) model G (Table 6), whose working is based on the movement of a weight attached to a spring. The gravimeter’s housing is metallic, which, although providing great strength, has the disadvantage that the structure will expand and contract as the temperature changes; therefore, the temperature has to be maintained constant. The sensor’s weight, unlike the rest of the structure, is made of quartz, which is less sensitive to temperature.

**Table 6:** Specifications of the LaCoste & Romberg gravimeter, model G.

LaCoste & Romberg (Model G)	
Range	7000 mGal
Data Resolution	0.005 mGal
Accuracy	0.04 mGal
Repeatability	0.01 to 0.02 mGal
Drift	1.0 mGal per month
Length	19.7 cm
Width	17.8 cm
Height	25.1 cm
Weight	3.2 Kg

The LCR-G is a relative gravimeter, that is, it does not measure gravity directly but instead provides a value for the difference in gravity between observations in different locations or in the same place but at different times.

Due to the influence of the weather on gravimeter measurements, a weather station is used to monitor weather conditions. The weather station development is a low-cost and low-power system based on the Arduino^TM^ [48] platform and consists of temperature, humidity, and pressure sensors (Table 7).

**Table 7:** Main components of the weather station.

Weather Station	
Component	Description
ATMEGA328	Microcontroller
BMP085	Pressure and Temperature Sensor
SHT75	Humidity and Temperature Sensor

The weather station sends the acquired data to the gravity meter system via a UART. The data is also stored locally on a memory card whose acquisition time is programmable.

The weather station’s two sensors are digital and communication is performed by a microcontroller, ATMEGA328, via one I2C serial protocol for the BMP085 and one pseudo I2C for the SHT75. After acquisition, data are stored in the on-board memory and are transmitted via the UART. Both sensors have built-in temperature sensors (Table 8).

**Table 8:** Specifications of the humidity and pressure sensors.

	Humidity Sensor		Pressure Sensor	
	Humidity	Temperature	Pressure	Temperature
**Resolution**	12 bits	14 bits	0.01 hPa	0.1 °C
**Accuracy**	±3%RH	±0.3 °C	±0.2 hPa	±0.5 °C
**Linearity**	±0.1%RH	±01 °C	--	--
**Range**	0%–100%RH	−4 to 123.8 °C	300 to 1100	−40 to 130 °C
**Offset**	<0.5 *RH/year	<0.04 °C/year	±1 hPa/year	--

#### 2.1.2. Measurement System for Ground Deformation

The sensor used for the ground deformation measurement system is the 701-2 tiltmeter from Jewell Instruments (Manchester, NH, USA) (Table 9). This high sensitivity and low power-consumption tiltmeter has two analog outputs corresponding to two axes (NS and EW) and a single analog output for a temperature sensor. This sensor is mainly useful in the field of geophysics for monitoring deformation of the Earth’s surface, although it can also be used for structure monitoring.

These tiltmeters contain two electrolytic level sensors, one for each axis, which produce a change in resistance in response to a rotation of the sensor. This resistance is measured in a Wheatstone bridge; the signal is then amplified so that it can be acquired externally.

The two sensors are arranged orthogonally such that the vector is the sum of the output of both channels given the direction and magnitude of the rotation relative to the gravity vector. The temperature sensor, installed inside the tiltmeter, evaluates how the temperature changes affect the structure in which the tiltmeter is installed.

**Table 9:** Specifications of the 701-2 tiltmeter.

701-2 Platform Tilt Meter	
Angular Range: low gain	±8000 urad (±0.46°)
Angular Range: high gain	±800 urad (±0.046°)
Scale Factor	1 urad/mV
Resolution	0.1 urad
Linearity	2% of full span
Tilt Output	±8 V (single-ended) and ±16 V (differential)
Temperature Output	0.1 °C/mV (single-ended) and ±0.75 °C accuracy

The default sampling rate of the tiltmeter is once a minute, although larger time intervals can be programed. However, the acquisition system takes samples every second and provides every minute the arithmetic mean of all samples. Thus, signal filtering is performed, which substantially improves the signal-to-noise ratio.

#### 2.1.3. Tide Gauge Measurement System

The sensor used for the ocean tide measurement system is the SEBAPuls 30 radar from SEBA Hydrometrie (Kaufbeuren, Germany) (Table 10). It is a non-intrusive sensor for high-precision measurement of water levels that functions by emitting microwave pulses of 26 GHz and receiving the waves reflected off the water surface. After receiving the reflected signal, the sensor calculates the distance to the water surface.

**Table 10:** Specifications of the SEBAPuls 30 radar.

SEBAPuls 30	
Precision	±3 mm
Range	0 to 35 m
Output	4 to 20 mA

#### 2.1.4. Monitoring System Using Images

This system was developed using a commercial camera (Table 11) designed specifically for the Raspberry Pi platform. It is a low-cost system that can be implemented on any of the above systems since its interface is independent from the others. The camera interface is Camera Serial Interface (CSI) a specification designed by Mobile Industry Process Interface (MIPI), an organization that develops interface specifications for mobile phones.

**Table 11:** Specifications of the camera.

Raspberry PI Camera Board	
Resolution	5 MP
Still Picture Resolution	2592 × 1944
Video	1080p @ 30fps, 720p @ 60fps and 640 × 480p 90fps
Interface	15-pin MIPI Camera Serial Interface
Size	20 × 25 × 9 mm

### 2.2. Software

The developed software consists of a set of programs written in C, PHP, HTML, Java, JavaScript, LUA, and Linux Bash shell scripts. These programs also use certain free software applications such as Gnuplot [49] for graphical representations of various parameters; ImageMagick [50] for editing images; libav [51] for video editing; Apache [52] as a web server; and the Telegram CLI [53] instant messaging program for controlling and configuring the various systems.

The execution of most of the programs are performed using *cron* of Linux, which is a background process manager able to run processes at regular intervals that can be scheduled according to the type of application.

The programs are divided into those related to data-acquisition systems (tiltmeter, tide gauge, and gravimeter) and the image-capturing system. The programs and scripts for the acquisition system are as follows: *main.c*, *graph.sh*, *synch.sh*, *meteo.c*, *message.lua*, and *index.php* (Table 12), while those used for the image-capturing system are: *timelapse.c*, *battery.c*, *bat_graph.sh*, *video.sh*, *buffer.sh*, *synch.sh*, *message.lua*, and *index.php* (Table 14).

**Table 12:** Data-acquisition system software.

Software/Script	Execution Every
main.c	1 min (default)
graph.sh	1 min
synch.sh	30 min
meteo.c	continuously
message.lua	continuously
index.php	continuously

Most of the programs in Table 12 have a short life cycle and once finalized are quit until they are next needed. In order to avoid that two instances of the same process have been executed, each time a program is launched it checks if another process already is running, in this case, that process is forced to finalize. A detailed description of these programs, corresponding to each data-acquisition system, is presented below.

**Figure 2:**
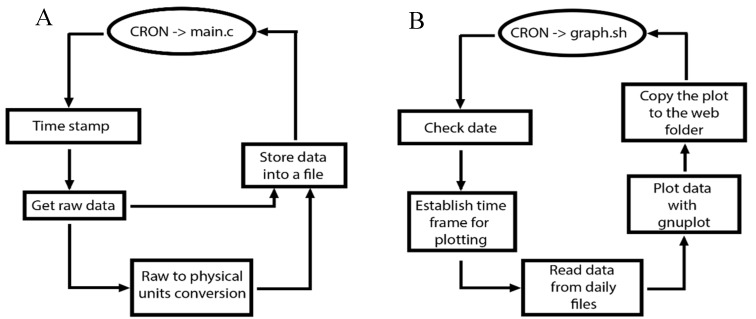
(**A**) Flowchart of the main.c application; (**B**) Flowchart of the graph.sh application.

The principal program is the *main.c* (Figure 2A), which performs the analog data acquisition. It acquires the data, timestamps it, and then stores it in an external USB memory. This memory has a structure of nested folders for each piece of equipment (gravity meter, weather station, tiltmeter, and tide gauge) containing subfolders for years and further subfolders for months, in which files are stored on a daily basis. This software uses the calibration curves of each sensor and stores the data in files for raw data and for calculated data corresponding to their units of measurement. The calibration curves are located in files inside the system and can be accessed by the acquisition software during each measurement.

Using the *synch.sh* script, which runs every 30 min, the stored data is fully and regularly copied to a remote computer, maintaining the same structure that exists in the external memory of the system. This periodic data-copying process is configurable, as is the server address to which the user sends the data. Data transfer is performed by applying Linux *rsync* over SSH protocol, which allows for the synchronization of local folders and files on a remote computer. The advantage of using this form of data transmission is that it minimizes the volume of data transmitted since it makes use of a delta encoding algorithm, that only stores the bytes that have been modified since the previous version of the file.

The *graph.sh* (Figure 2B) script is the visual representation of the data that are acquired that uses the Gnuplot application tool. This is useful for generating graphs from the command line that are stored directly as a file (pdf, png, gif, jpg, *etc.*). This script runs once a minute and its function is to take data from a specific period and represent them using Gnuplot. The output of this software, a PNG image, is copied to the path where the website is hosted. The period of data to be displayed is configurable by the user and before presenting any visual representation this script reads a configuration file containing the chosen period.

Both the computer settings and the display of real-time data are carried out through a web page developed in PHP (Table 12). This website is password-protected to prevent modifications of the system configuration by unauthorized users. The sections that must be configured through the webpage are: station name, serial number of the sensor, selection of the channels to be acquired, and the sampling period. If the user wishes to transmit data, the transmission section must be activated and the server must be configured to specify the port and the remote folder in which the data should be saved. Optionally, the deployment coordinates (latitude, longitude, and altitude) can be added to the station. Other settings such as the calibration curves of the sensor are not performed directly through the website for safety reasons. The user must access the system via SSH and modify the corresponding file. If the user does not want the data transmitted in real time, they can be downloaded directly from the website.

**Figure 3:**
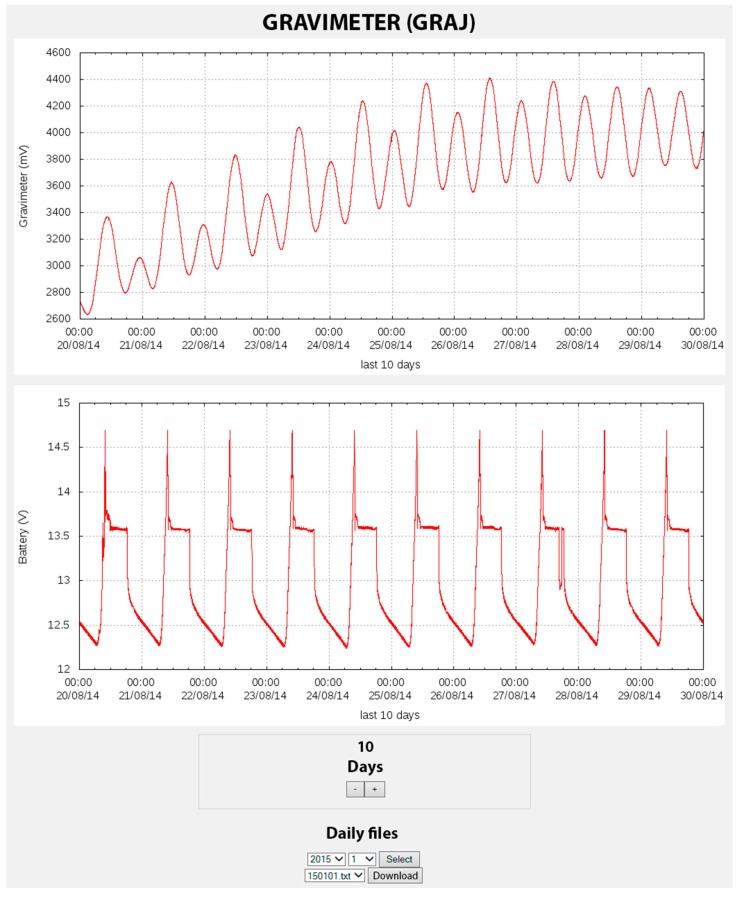
Image captured from the website corresponding to gravimeter measurements and battery control with the units of the depicted parameters in mV and V, respectively. A 10-day period is shown.

**Figure 4:**
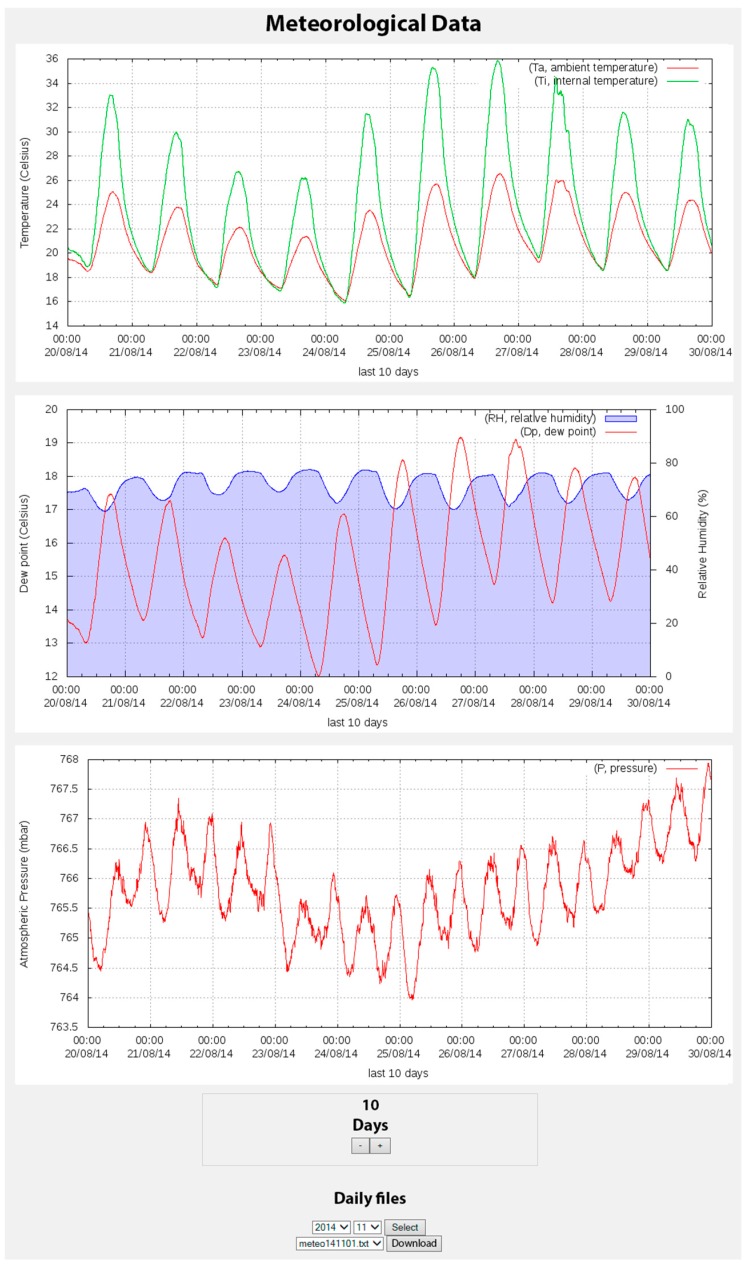
Image captured from the website corresponding to the meteorological data of the gravimeter system. Top: the ambient and internal temperature; middle: the relative humidity and dew point; bottom: the pressure. A 10-day period is shown.

**Figure 5:**
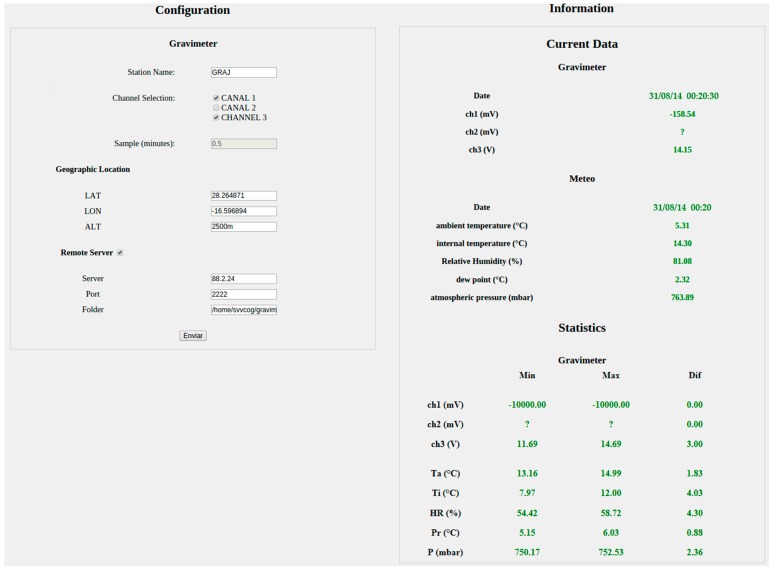
Image captured from the website corresponding to the gravimeter configuration section and metadata. Left-hand side: the configuration data; right-hand side: information regarding the gravity meter system, battery, and meteorological data.

The website information is presented both numerically and graphically. The graphic representation of the data is carried out for all parameters measured on the previous days (Figure 3 and Figure 4). Moreover, it provides information regarding the acquired data such as date, time, and the most recent value, and also provides a brief statistical summary of the data for the current day (minimum, maximum, and the difference between them) (Figure 5). The configuration files generated by the website are used in the scripts described above.

As explained above, the developed applications are executed by the system at scheduled intervals; however, there are other programs such as those for acquiring meteorological data and the instant messaging application that run on system start-up.

**Figure 6:**
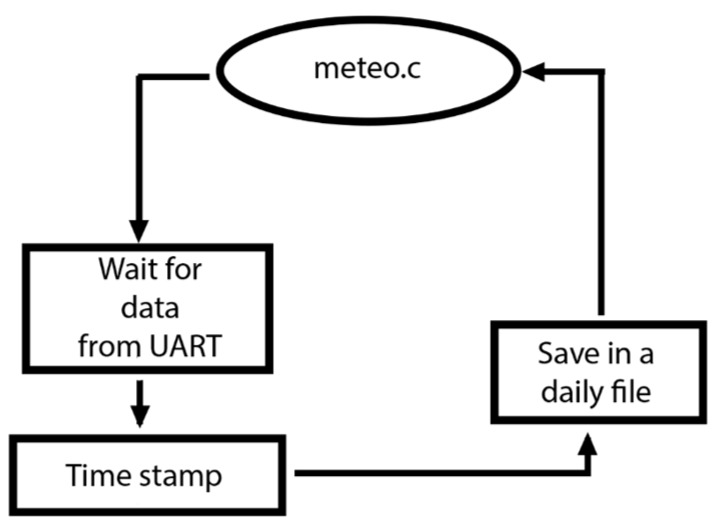
Flowchart of the meteo.c application.

The data generated by the weather station are sent from the Arduino^TM^ platform via the UART every minute. The *meteo.c* program handles data reception, provides the timestamp, and then stores data on the USB drive in the file structure described above (Figure 6).

**Figure 7:**
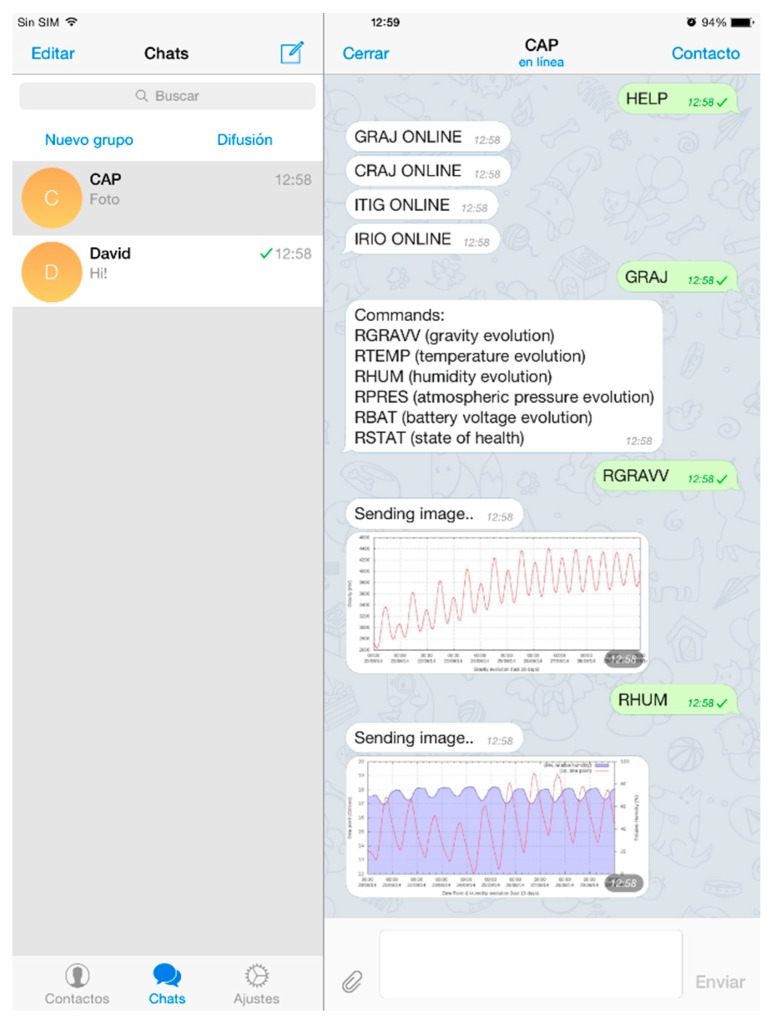
Querying data from various stations in real time via the Telegram instant messaging application. First, the status of the stations is requested by the general command HELP and then the station to be accessed—in this case GRAJ—is specified. After receiving this command, the station will reply with the rest of the available commands supported by the station. In this example, the graph of gravity and humidity for the previous ten days has been requested.

The other application that runs at system start-up is the Telegram-CLI instant messaging service, an unofficial client telegram [54] for Linux. Using scripts in *Lua* language, users can interact with the messaging software and send and receive text, images, and video. This enables the status of the various pieces of equipment to be checked and configured. The *message.lua* script (Table 12) acts as a robot, answering via the messaging program commands sent by users. The commands requesting information from the station can be images, video, or text. They are received by all stations but, because each station measures different parameters and is installed in a different location, each has a specific script according to the parameters measured. For each computer connection, there is a general HELP command, executed by all, in which the response is the name of the station followed by ONLINE. To find out which commands are supported by each station, it is only necessary to type in the station’s name (Table 13). An example of how to access various stations via instant messaging software is shown in Figure 7.

**Table 13:** Some of the commands supported by the stations. The general commands are answered by all stations.

Command	Type	Response	Station Responding
HELP	General	<station name> ONLINE	All
<station name>	Specific	Lists the commands supported by the station	All
CRAJI	Specific	Sends the last image captured	Camera CRAJ
CRAJL	Specific	Sends a list of available time lapses	Camera CRAJ
RGRAVV	Specific	Sends the graph for gravity	Gravimeter GRAJ
RBAT	Specific	Sends the graph for batteries	Gravimeter GRAJ
ITIGXY	Specific	Sends the graph for deformation	Tiltmeter ITIG
IRIOXY	Specific	Sends the graph for deformation	Tiltmeter IRIO

The software for the image monitoring system works in a similar way to other acquisition systems, with a number of programs being executed by *cron* and others running on system start-up (Table 14).

**Table 14:** Imaging-monitoring system software.

Software/Script	Execution Every
timelapse.c	1 min (default)
battery.c	1 min
bat_graph.sh	1 min
video.sh	Once every day
synch.sh	1 min
buffer.sh	Once every day
message.lua	Continuously
index.php	Continuously

**Figure 8:**
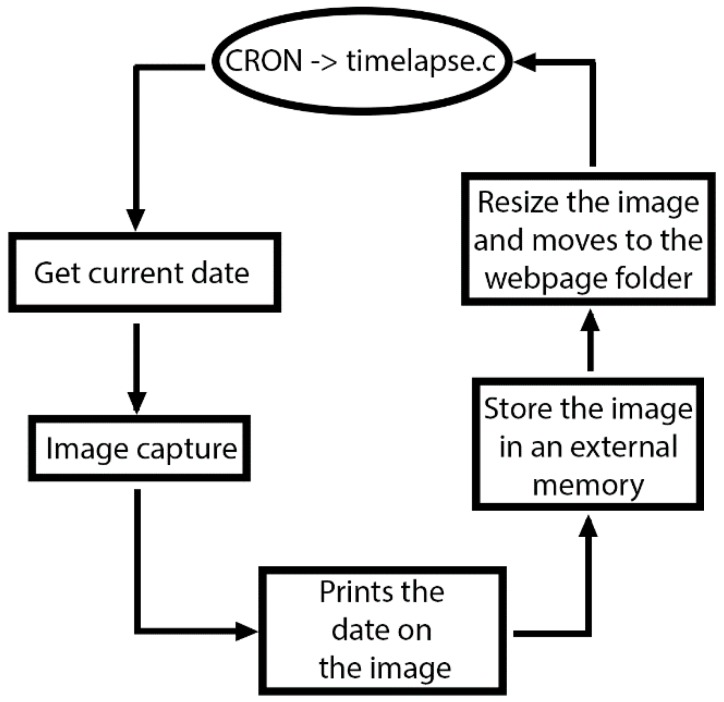
Flowchart of the timelapse.c application.

The *timelapse.c* application captures images: when running, it records the current UTC time, captures the image, and prints the time on it. Next, it saves the image to a USB external memory, before reducing its size and copying it to the route of the website (Figure 8).

The *sync.sh* script saves images to a remote server, as explained above.

The *buffer.sh* script, executed once a day, verifies that the size occupied on disk by the images does not exceed the memory capacity. It deletes old images, thereby establishing a circular image buffer.

The *video.sh* script, executed once a day, records a video montage of all the images from the current day and stores it in the memory. It makes two videos, one of low quality to be downloaded via mobile devices and the other of high quality that can be downloaded from the website.

Given that the system is based on the diagram depicted in Figure 1, one of the analog channels is used to control the system’s battery. This control is carried out by *battery.c* software that continuously acquires and stores the battery value in the external memory.

The *bat_graph.sh* script creates a graphical representation of the data acquired for the battery and backs it up to the route of the website, following the flowchart illustrated in Figure 2B.

**Figure 9:**
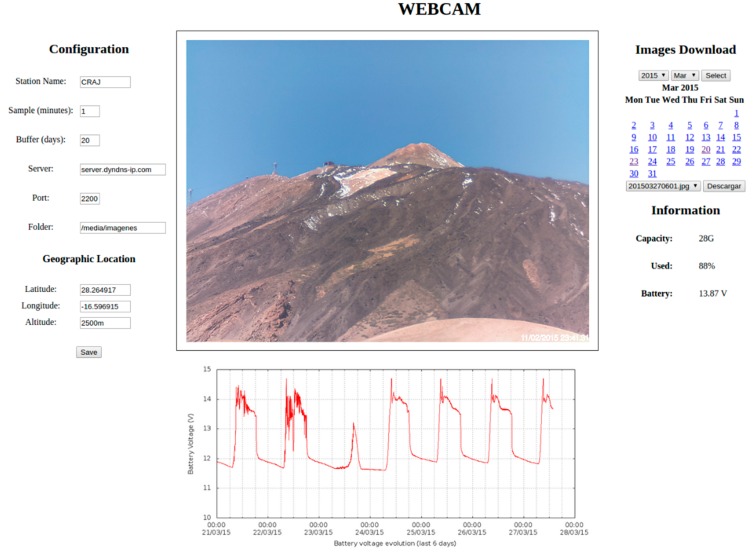
Left-hand side: the system configuration; center: the most recent picture of Teide Volcano (Tenerife) taken from a distance of approximately 4 Km and a graph of the evolution of the battery; right-hand side: information about the system, as well as the option for downloading images using a calendar.

The configuration of the system and other station information is accessed via a website developed expressly for this purpose (Figure 9). The sections that have to be configured via this website are as follows: station name; image shooting rate; buffer size of storage; server address and port; name of folder on the remote computer if the backup is activated; and the geographical location of the station. In addition, the information about the station provided on the website contains the following: previous image taken by the camera; a chart with the evolution of the battery voltage over the previous six days; external memory capacity; percentage of this memory used; and current battery voltage. As well, the high-resolution images and the videos generated can be downloaded from the website.

The images are taken at programmable time intervals of 1–59 min. If desired, they can be sent to a remote computer or downloaded from the website. At the same time, the system acquires and draws a graph with the battery voltage for every minute and updates the information regarding the device’s capacity. The system can operate in three different ways: taking pictures continuously during the day and night, using a light sensor in order to detect the daylight, and using a sunrise/sunset table, which is the default mode. By the end of the day it generates a time-lapse which can be downloaded from the website.

**Figure 10:**
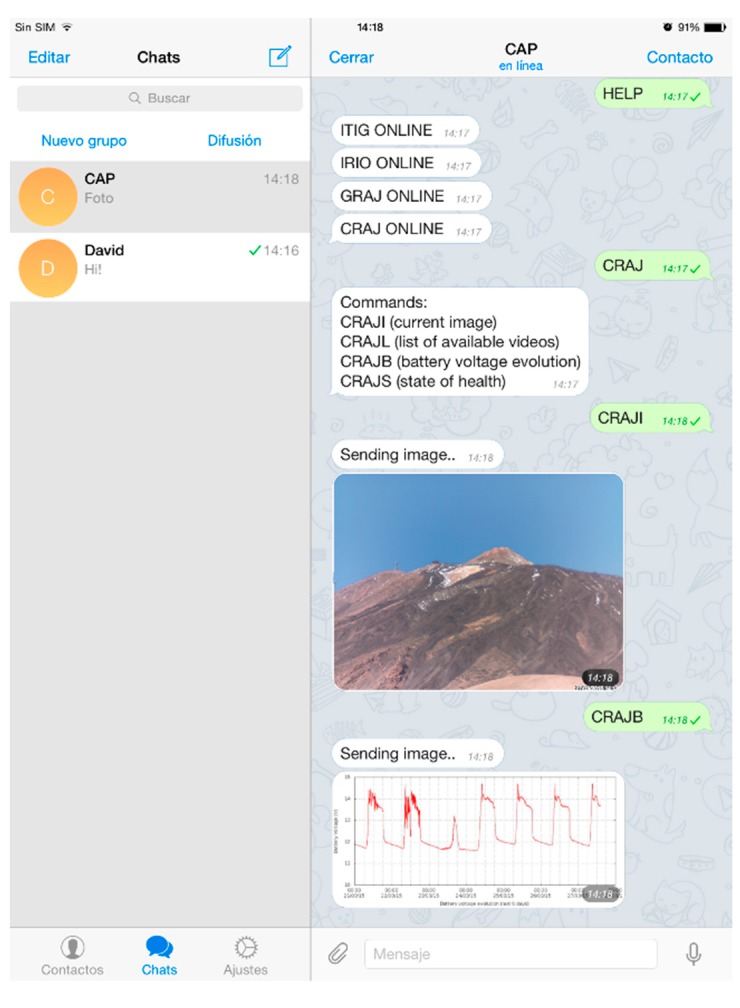
Querying the seasons via the Telegram instant messaging software using a smartphone. The last picture taken by the CRAJI command is sent and the battery status is checked with CRAJB command requests.

In an application such as this, which monitors a site via images, instant messaging software plays a very useful role since, via the use of commands, the latest image or a video consisting of all the pictures taken during the day can be downloaded as a very easy and quick daily review of all the images (Figure 10).

## 3. Results and Discussion

**Figure 11:**
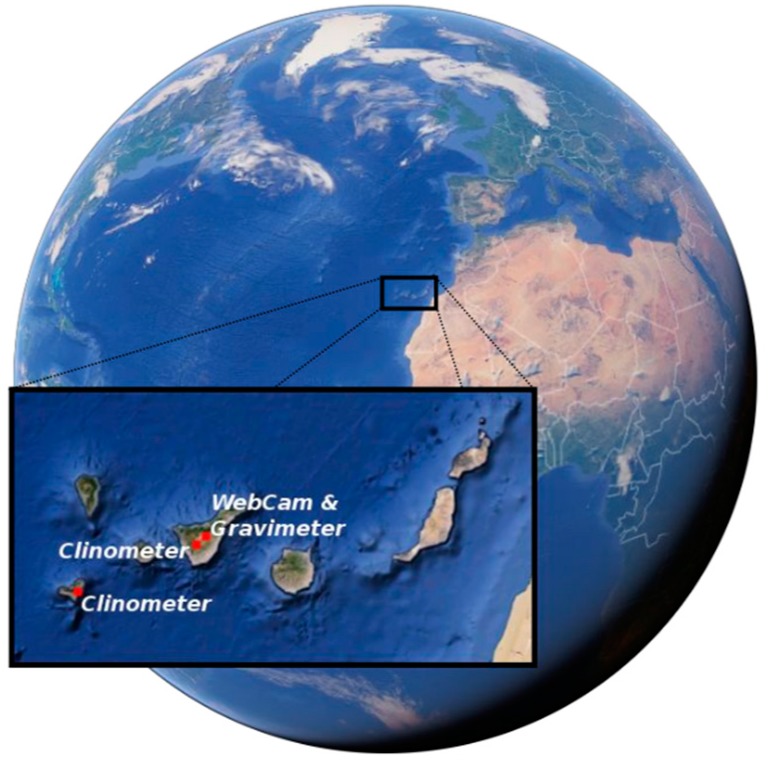
Location of the four different measuring systems.

The systems described in this paper are installed and working in different parts of the Canary Islands (Figure 11). Three of the systems are installed on Tenerife and one on El Hierro.

### 3.1. Gravity Measurement System

**Figure 12:**
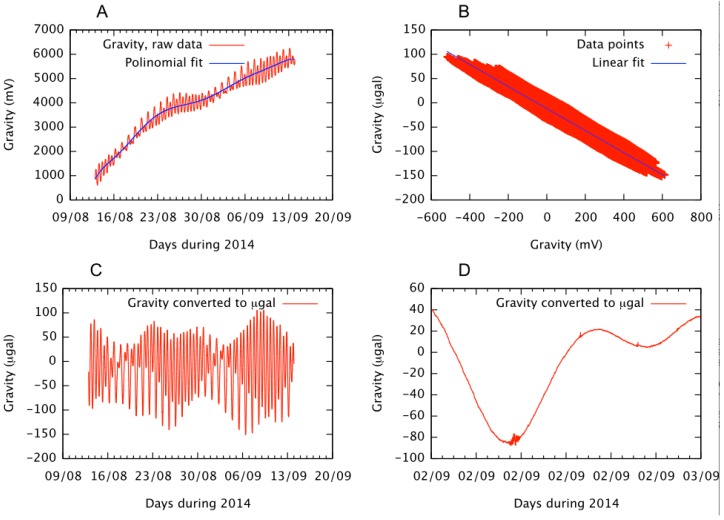
(**A**) Graph of the registered gravity and the trend curve; mV are plotted against days; (**B**) Graph of the adjustment of the line of the points obtained from the theoretical gravity (µgal) and experimentally obtained values (mV); (**C**) Graph of the gravity converted to µgal; (**D**) Detail of the previous graph in which the effect of a distant earthquake is registered.

The gravity measurement system has been operating since 6 August 2014 in Las Canadas del Teide, near the Teide-Pico Viejo complex (28°15′35.5″ N, 16°35′48.8″ W). The system was installed with good thermal insulation to avoid errors in the gravity measurements. To check that the system is operating properly, the gravity measurements were compared with the theoretical gravity curve and the system was calibrated accordingly. The theoretical gravity curve is obtained from theoretical land- and ocean-tide loading at the point where the instrument is installed (Figure 12).

The developed system has a drift over time fitting an eighth grade polynomial that is due to the effects of temperature, humidity, and pressure, as well as to the drift of the measuring device itself (Figure 12A). This drift correction is performed by subtracting the polynomial from the data recorded.

The conversion factor given by the manufacturer of the sensor allows for the conversion of data from mV to µGal. Knowing the theoretical curve of gravity, the conversion factor can be obtained and adjusted with an equation (mx + b) using the least squares of the points obtained by comparing the theoretical signal (µGal) to the actual signal (mV). This gives equation parameters of −0.227 m −11,831, with a mean square error of 0.97 (Figure 12B). The gravity in µGal without instrumental drift is given below (Figure 12C).

The low signal noise (less than 1 mV), along with the high sensitivity of the equipment, enables signals associated with distant earthquakes to be registered (Figure 12D).

### 3.2. Ground Deformation Measurement System

Two ground deformation measurement systems were installed in galleries built for water extraction: one on Tenerife (28°14′50.3″ N, 16°43′16.4″ W) on 17 June 2014 and one on El Hierro (27°47′18.2″ N, 17°55′19.9″ W) on 11 June 2014 (Figure 11). These systems were installed in underground galleries—sites that guarantee high thermal stability—due to their sensitivity to temperature changes. In addition, the installed systems were thermally insulated to minimize the possible effects of sudden changes of temperature.

**Figure 13:**
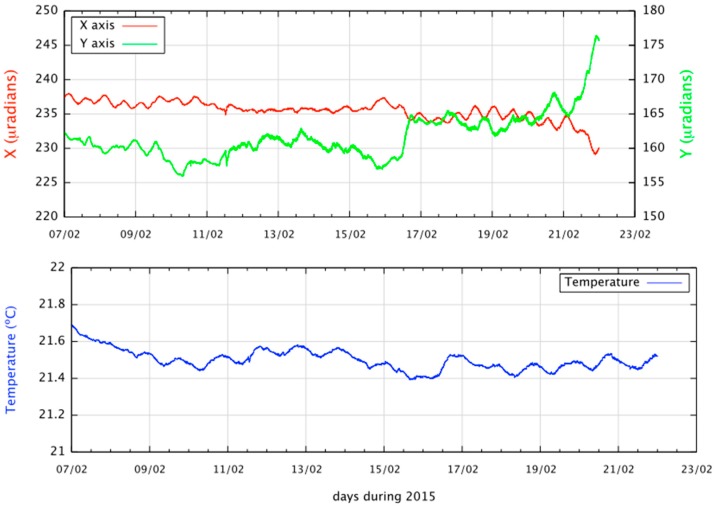
Fifteen days of data from the ITIG station on El Hierro. The upper graph represents the ground deformation observed for two perpendicular axes in µRad. The bottom graph shows the temperature in degrees Celsius.

The system has high thermal stability (Figure 13) and temperatures do not vary more than 0.2 °C, which is of great important since the response of these sensors is highly correlated to temperature. The sensitivity of these systems (over 0.1 µ*R*ad) reveals a ripple signal at the top of the figure, corresponding to ground movement caused by the Earth’s tides at the site in which the sensor is installed.

**Figure 14:**
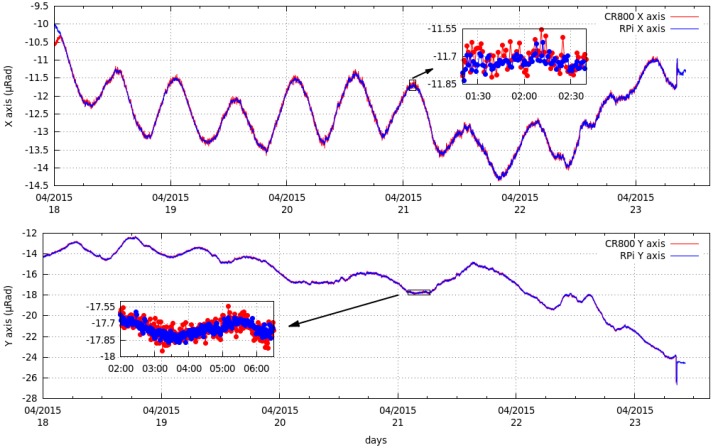
Comparison between a commercial system and the developed system. The upper picture depicts the evolution of the X-axis and a detail of the noise in both systems. The graph below is the same for the Y-axis.

A comparison between a commercial acquisition system (Figure 14), CR800 from Campbell Scientific [55], and the system developed in this work was performed. Given that this commercial datalogger is a 12-bit system, its input was adjusted to ±2500 mV to obtain a resolution as close as possible to our system. Our sensor, whose output in differential mode is ±16 V, had to be zeroed very accurately so that its evolution during the days that the comparisons were carried out did not exceed the input range of the commercial system.

The evolution of both systems (Figure 14) was the same but the noise in the developed system was one magnitude below that of the commercial system (Table 15). This is partly because the commercial system has a resolution of 12 bits that can measure a range of ±2.5 V, while our system has a resolution of 16 bits that can measure a range of ±8 V (unipolar). Despite the differences in the data obtained, this comparison provides a indication of the quality of the developed system.

**Table 15:** Noise measured in both acquisition systems.

	CR800	RPI
X axis	0.15 µRad	0.047 µRad
Y axis	0.20 µRad	0.03 µRad

### 3.3. Tide Gauge Measurement System

The tide gauge measurement system has been in operation since 29 November 2013 in the port of Los Cristianos in the south of Tenerife (28°02′51.2″ N; 16°43′07.3″ W), where it records ocean tides in parallel to two other commercial systems, *Vegamet* 381 [56] and *LogoSens* [57].

**Figure 15:**
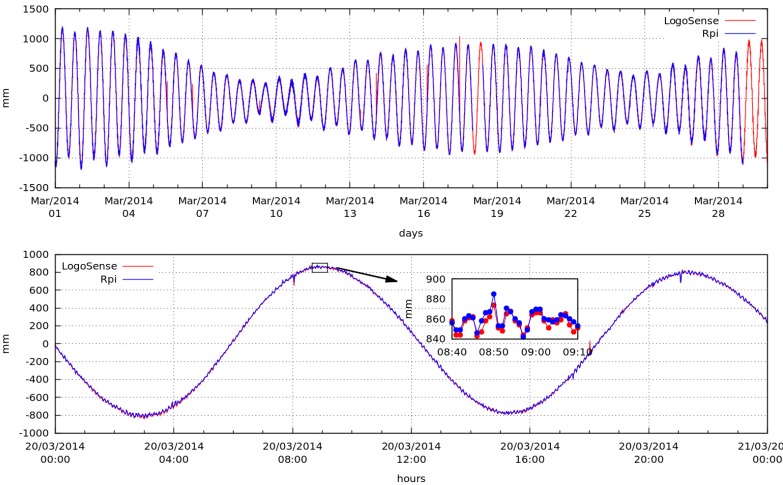
Ocean tide registered by a commercial acquisition system and by the system developed in this project over a period of thirty days. Top: the measurements obtained by the LogoSens datalogger (in red) and the measurements produced by the datalogger developed in this project (Rpi) (in blue). Bottom: the same signal over a period of 24 hours with a zoom showing the noise present in both systems.

The signal depicted in this image corresponds to a complete cycle of 28 days. In the image, the spring tides, when the Moon is aligned with the Sun, and neap tides, when the Moon, Sun and Earth form a 90° angle, can be appreciated (Figure 15), with measurements taken by the *LogoSens* commercial system and by the system developed in this work. The inset corresponds to a detail of the signal noise, which is the sum of the electronic noise and the noise produced by the waves (given that the measuring system is not stationary on the surface of the sea). The noise resulting from adding the electronic contribution and waves is less than 2 cm. This noise is greatly reduced if the systems implement the same process as in the tiltmeter system, in which the measurements are calculated every minute by averaging samples taken every second. In this case, the design specifications enable this system to perform these calculations.

### 3.4. Imaging Monitoring System

**Figure 16:**
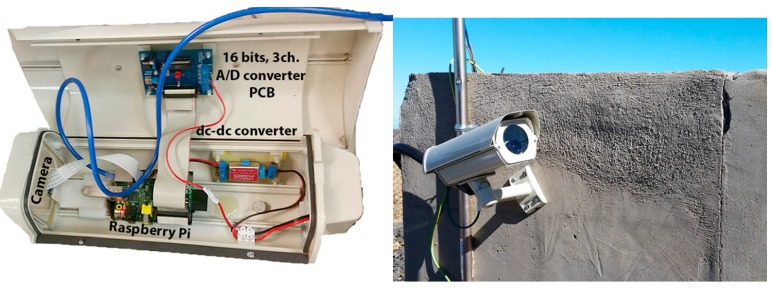
Imaging monitoring system.

The imaging monitoring system has operated for over a year in a number of different geographical locations on Tenerife, and is currently installed in its final location on the Rajada mountain (28°15′53.1″ N, 16°35′48.6″ W). These test sites was used to check its performance under different weather and light conditions. Due to its altitude, the monitoring area has a mountain climate, with regular snowfall and very low temperatures in winter. Moreover, during the day, the intense light can affect the quality of the images. The system has been running fault-free at Rajada since 20 August 2014. Each day, it generates 1.3 Gbytes of images, even though each image is compressed and no images are taken at night (Figure 16). Currently, a similar system is being prepared for installation in the crater of the Teide to perform visual monitoring of this volcano’s fumarole field. The great advantage of using this system is its low cost compared to other cameras that offer similar features.

## 4. Conclusions

This article describes the development of a general-purpose and low-cost monitoring system that can be used in volcano monitoring. It is very versatile and can accommodate different sensors for developing stations for monitoring gravity and ground deformation, and for imaging monitoring. In addition, it gives a high-quality signal-to-noise ratio. Its versatility lies in its use of up-to-date hardware and software that facilitate the development of applications in an Internet environment. It uses an embedded Linux platform and can run diverse applications written with some of the many high-level languages available for this platform.

Instant messaging has evolved for mobile applications, cross-platform applications, and even Web Services and does not require any application. This is the case of instant messaging applications such as Facebook Messenger, Skype, Whatsapp, and Telegram. The system presented here make use specifically of Telegram to communicate via any device (PC, smartphone, or tablet). Users can access the developed system at any time via the instant messaging software and retrieve information about the overall state of the system and its batteries, and visualize graphs with data collected from a specified sensor or a photo.

The system configuration can be done via a web page that is password-protected to restrict access and to prevent data loss. From this website, users can also perform a general check of the system status and view data in real time. Compared to other existing systems, the main advantage of the system we present here is its low cost and homogeneity, which ensures that maintenance is simple.

Given that the manufacturers of different acquisition systems aim to meet users’ needs, products are becoming more complex and now incorporate many types of communication systems, as well as many analog and digital channels to be used with any type of sensor. This increases the cost of products (Table 16). Our system, on the other hand, employs simple but effective electronics, and the development of various applications for data exploitation is facilitated by the use of the Linux working environment. Aside from the low cost of the components, the overall advantages of this system are obvious.

It is an open system and all the software used is free. It is a low-noise system and is able to measure small variations in parameters with only 2 bits of error in its 16-bit resolution. This means that for applications such as gravity measurement, the expected noise is 1 mV or 0.2 µGal (Table 13). It also has good power performance and consumes approximately only 200 mA when running at 12 V. As well, taking into account its Internet connectivity and low power consumption, it can be run on solar panels and be easily installed in remote inaccessible areas. In that case, a radio link must be used if it is desirable to have a permanent connection with the system, which is the case of volcano monitoring, and the power consumption will depend on the radio link used. For example, the consumption of a system with a camera and a WIFI link at a distance of 5 km is of the order of 400 mA. This versatility facilitates its installation in areas of great interest for volcano monitoring such as underground galleries, where the system benefits from high thermal stability with variations that do not exceed 0.2 °C (Figure 13). Its low cost—less than 10% of the cost of other commercial dataloggers—means that maintenance is simpler as many units can be purchased (Table 16). Table 17 shows a summary of the advantages of the Volcanic Monitoring system built and introduced in this paper.

**Table 16:** Commercial dataloggers and designed datalogger.

Product	Description	Price
DT-85G (dataTaker)	48 Analog I/O, 4 Digital I/O. RS232/485, ETH, USB MODBUS RTU/TCP	4000 €
DT-80 (dataTaker)	15 Analog I/O, 12 Digital I/O. RS232/458, ETH, USB MODBUS	3000 €
R800 (Campbell Scientific)	6 Analog I/O, 4 Digital I/O. RS232	1000 €
Our datalogger	Raspberry Pi, Raspberry Pi Camera, A/D 16 bits, RTC, PCB Arduino UNO + Sensors	156€

Currently, all the stations using this application are integrated into the Canary Islands volcano monitoring system belonging to the Spanish National Geographic Institute.

**Table 17:** Advantages Summary.

General-purpose volcano monitoring system (gravity, ground deformation, imaging monitoring)
Low-cost volcano monitoring system (156 €)
Simple maintenance service
High-quality signal-to-noise ratio (gravity measurement noise is 1 mV or 0.2 uGal)
Open system where all the software used is free
Development of applications in an online environment
Use of Linux working environment
Good power performance (200 mA at 12 V)
